# A Sentence Repetition Task in Spanish language: a valid tool for early language assessment

**DOI:** 10.1590/2317-1782/20232022164en

**Published:** 2023-09-15

**Authors:** Natalia Bravo Cerro, Miguel Lázaro López-Villaseñor, Irene Rujas Pascual, Sonia Mariscal Altares

**Affiliations:** 1 Departamento de Pedagogía, Facultad de Ciencias de la Salud, Universidad de Castilla la Mancha - UCLM - Talavera de la Reina, Toledo, España.; 2 Departamento de Psicología Evolutiva y de la Educación, Facultad de Psicología, Universidad Nacional de Educación a Distancia - UNED - Madrid, España.; 3 Departamento de Psicología Experimental, Procesos Cognitivos y Logopedia, Facultad de Psicología, Universidad Complutense de Madrid - UCM - Pozuelo de Alarcón, Madrid, España.; 4 Departamento de Investigación y Psicología en Educación, Universidad Complutense de Madrid - UCM - Pozuelo de Alarcón, Madrid, España.

**Keywords:** Developmental Language Disorder, Early Language Assessment, Grammatical Development, Sentence Repetition, Specific Language Impairment, Nonword Repetition Task

## Abstract

**Purpose:**

Sentence Repetition Tasks (SRT) have been widely used to assess early language abilities in different languages and populations. In addition, it has been proved that performance in SRTs serve as a clinical marker to detect language difficulties. However, most of the research has been conducted in English language and with children older than 4 years of age. Despite this scarcity, [1] developed a SRT for monolingual Spanish-speaking children between 2 and 4 years of age. Initial findings showed that it is a useful tool for discriminating children with different linguistic levels. In addition, the task showed concurrent validity with a nonword repetition task. In the current study we want to explore the predictive validity of this task.

**Methods:**

We conducted a longitudinal study including 20 monolingual Spanish-Speaking children who were tested twice, at 33 months of age and six months later. In addition to the SRT, participants completed a nonword repetition task [2] and the Spanish version of the Merrill-Palmer-R Developmental Scales [3].

**Results:**

showed strong and positive relationships between the different tests when first assessed. We also found strong and predictive relationships between the SRT at time 1 and SRT and the Merrill-Palmer-R at time 2.

**Conclusion:**

We conclude that the SRT developed [1] is a valid tool for examining early language abilities and its changes over time.

## INTRODUCTION

One of the most interesting but difficult challenges for clinicians and researchers nowadays is related to the assessment of early language development. It is important to go beyond a mere description of the milestones in the process and get to the heart of how do the different mechanisms responsible for language acquisition work. In order to achieve this aim, it is necessary to develop simple and reliable tools to assess very young children^(1,2,3)^. Early assessment is essential for early detection, and early detection allows a sooner intervention. As it has already been stated in the literature that an early intervention increases the chances of success, early identification of language difficulties entails better chances for successful prevention of early developmental risks^(4)^. 

In the last decades, Sentence Repetition Tasks (SRTs) have been widely used as tools to detect children with Developmental Language Disorder (DLD)^(5)^. Children with DLD present important difficulties in both expressive and comprehensive abilities. Research has suggested that SRTs allow the assessment of linguistic mechanisms that underlie these abilities and, thus, seem to be sensitive tools to detect children with these linguistic characteristics^(6-9)^.

In SRTs, children are asked to immediately repeat aloud several sentences that the examiner orally presents. It may seem a simple task, but it has been stated that different processes at phonological, morphosyntactic and semantic level are required in order to succeed. At the same time, children must be able to store and retrieve verbal information^(10)^. This means that both linguistic and memory systems must work together to provide a correct answer^(11,12)^. In addition, research has suggested that language comprehension is also involved, as comprehending the meaning of the sentence implies the development of mental representations that increase the probability of a correct repetition^(13)^.

Recently, SRTs have been proved to be useful tools to detect DLD in both monolingual (see, for example, Pham and Ebert^(14)^, for Vietnamese speakers) and bilingual populations (see, for example, Fleckstein et al.^(15)^, for Arabic-French and English-French-speakers; or Gavarró^(16)^ for Spanish-Catalan-speakers). In addition, it has also been used to detect language difficulties in children with Dyslexia, given that sentence repetition abilities are related to further language and reading skills^(12)^. It is important to keep examining this relationship, as there is increasing evidence suggesting that a high percentage of children with Dyslexia present DLD too. This comorbidity is under diagnosed, entailing big problems when planning an intervention^(17)^.

At the same time, there is plenty of research showing that nonword repetition is also a good clinical marker for DLD^(6,18)^. Nonword repetition tasks (NWRs) have been successfully administered both in monolingual^(6)^ and bilingual populations^(19,20)^; and in low socio-economic status and culturally diverse settings^(21)^. NWRs provide more specific information related to the phonological and lexical knowledge of a language^(10,22)^. Instead, SRTs additionally provide information related to semantic and morphosyntactic development. This turns SRTs into more general and complete assessment tools^(8)^ with greater sensitivity and specificity, compared to nonword repetition^(23)^.

Anyway, both tasks have been proven to be useful tools to collect information related to linguistic skills in young children and to early detect language difficulties. Considering the supporting evidence of the SRTs as clinical markers of DLD, sentence repetition has turned to awaken the interest of clinicians and speech therapists that work with children. However, still today, most of the research has been carried out with children older than 4 years of age, leading to a lack of studies with children under 4^(24)^. In one of the few studies with younger children^(25)^ they administered a SRT to typically developing Italian-speaking children between 2 and 4 years of age. They found that the scores in the SRT allow to differentiate young and older children when assessing morphosyntactic development. Additionally, they found a relationship between sentence repetition, mean length utterance (MLU) in free speech and verbal memory. Children with larger MLU scored higher in the SRT compared to children with shorter MLU. Recently^(26)^, designed a SRT in Hebrew to evaluate two groups of children with and without hearing impairments, between 22 and 40 months. These authors found a high variability in both groups without significant differences between them, showing that the morphosyntactic ability remains intact in children with hearing impairment in these early ages.

In the case of Spanish language there is a lack of studies that use the SRTs as a tool for early linguistic assessment (see Rujas et al.^(24)^ for a scoping review). Most of the research conducted in Spanish language in the last decade has involved samples of children older than 5 years old^(27)^; samples including bilingual children^(16,28)^ or samples including children with difficulties and impairments^(29)^.

To our knowledge no study, other than^(1)^, has developed a specific sentence repetition task to assess morphosyntactic abilities in typically developing Spanish-speakers younger than 4 years old. In this research^(1)^, designed a 33-sentence repetition task considering several variables such as the frequency of the words selected, the syllable structure, and the phonological composition of these words, as well as the morphosyntactic complexity of the sentences. It was administered to 130 Spanish-speaking children aged 2 to 4 years old. The results of this study offered a Cronbach´s coefficient alpha of .971 and an inter-rater reliability of Kappa=.84. This task also allowed the authors to discriminate participants according to their age (older children -3 to 4-, performed better than the younger ones -2 to 3 years old).

Considering the scarcity of studies and the clinical need for well-designed instruments for early language assessment, we carried out this study in order to deeply explore the properties of the Spanish SRT^(1)^ as a measure of early morphosyntactic development. To this aim, we designed a longitudinal study including children aged 2 to 4 years old, to test its predictive validity, examining the extent to which performance in this task can predict future performance in the same or similar tasks.

## METHODS

### Participants

Twenty children (11 girls and 9 boys) participated in this longitudinal study. Children’s age ranged from 24 to 42 months at Time 1 (T1) and from 30 to 48 months at Time 2 (T2), six months later. Table 1 shows the data related to the age.

**Table 1:** Participant’s age (in months) at T1 and T2

	N	Minimum	Maximum	Mean	Standard deviation
Age **T1**	20	24.00	42.00	33.05	5.15
Age **T2**	20	30.00	48.00	39.90	5.04

Participants were recruited in different childcare centers in the cities of Madrid and Toledo (Spain). Their families (medium socioeconomic status) completed a consent form. All children were monolingual Spanish speakers and Spanish was the only language spoken at their homes. None of them presented any hearing loss or had required any speech therapy.

## MATERIALS

### Sentence Repetition Task (SRT)

The Spanish Sentence Repetition Task^(1)^ was administered to all children. This task includes 33 sentences that vary in length and morphosyntactic complexity (See Appendix A). The SRT was individually carried out in a play context. Following the procedure of^(1)^ the examiner created a story of a puppet that has not yet learned to talk and invites the participant to help her teach the puppet how. The examiner orally presents the sentences, one at a time, and the children are asked to repeat them aloud. Every 5 or 6 sentences, the examiner provides the participant with a sticker that he can paste on a cardboard with the drawing of a train.

### Nonword Repetition Task (NWR)

The Spanish Nonword Repetition Task developed by Mariscal and Gallego^(2)^ was also administered. This task consists of a list of 18 words and 18 nonwords. The task was, again, individually carried out in a play context. The pretext was the same (helping a puppet how to learn to talk). Words were first orally presented. After a short break, nonwords were presented too. Children were asked to repeat them aloud. Only the list of pseudowords was considered for this study.

### Merrill-Palmer-R Scales of Development

The Spanish version of the Merrill-Palmer-R Scales of Development^(3)^ was administered. These scales allow the individual assessment of children up to 6 years of age. They have been widely used by researchers and clinicians, as they explore different areas in development: cognitive, communicative and linguistic, motoric, socio-emotional and behavioral. For this study, we administered the expressive language subscale that includes the examiner assessment. The examiner compiled the information from the participant in a booklet that provided a raw score.

The research was approved by the Research Ethics Committee (CI-101-1896) and all the caregivers involved have signed the Free and Informed Consent Form.

### Design and procedure

We carried out a longitudinal study. Participants were assessed at two different moments (T1 and T2) separated by 6 months. As it has already been mentioned, the examiner individually assessed the participants in a quiet room, separated from the rest of the group. First, the Merrill-Palmer-R scale was administered, followed by the SRT. The NWR was presented last.

### Transcription and coding

Both the SRT and the NWR were audio recorded for later transcription. Sentences and words were orthographically transcribed. Coding was carried out as follows:

### Sentence repetition task

For this study we calculated (both at T1 and T2):

- Performance / accuracy in the SRT: it refers to the total number of sentences (out of 33) that the participant correctly repeats. Every correct sentence scored 1. Sentences that were not fully correctly repeated scored 0. Thus, participants could achieve a total score between 0 and 33.
- Number of omissions: it refers to the number of words that are omitted when repeating the sentence.

### Nonword repetition task

Following^(2)^ we coded phonological accuracy. Participants scored 1 for every correct repetition and scored 0 for every incorrect repetition. Thus, participants could achieve a total score between 0 and 18.

For both tasks, SRT and NWR, phonological errors were not penalized. That is, we considered the data relative to typically phonological development in Spanish language^(30)^ and did not penalize the errors that can be considered as part of the typical learning process (see Chart 1).

**Chart 1:** Types of errors permitted in the SRT and the NWR task

**TYPE OF ERROR**	**EXAMPLE IN SPANISH LANGUAGE**
Cutting down groups of consonants.	*Tren → Ten*
Substituting /r/ for other consonants.	*Nariz → Nadiz*
Omitting /r/, /s/, /θ/ consonants in the final position.	*Luz → Lu*
Substituting /θ/ for /s/ or /f/.	*Pez → Pes*

### Merrill-Palmer-R scales of development

The examiner scoring in the Language Scale was administered. This scale provides information related to the expressive language skills of children. We considered the direct scores obtained in this scale. Thus, the minimum score that could be obtained was 0 and the maximum score was 36.

### Research questions

The current study addresses the following research questions:

**RQ 1:** Is the SRT sensitive to the changes in performance (accuracy and omissions) from T1 to T2?
**RQ 2:** Does performance in the SRT at T1 predict performance in the SRT at T2?
**RQ 3:** Is performance in the SRT at T1 related to performance in other language measures, such as the NWR and the Merrill-Palmer-R at the same time?
**RQ 4:** Does performance in the SRT at T1 predict performance in a standardized language measure such as the Merrill-Palmer-R at T2?

## RESULTS

### Is the SRT sensitive to the changes in performance from T1 to T2?

Results show that children performed significantly better at T2 compared to T1; t(19) = -5.49, *p =* .001. That is, at 33 months of age, children can correctly repeat 15.70 sentences (out of 33). Six months later, participants can correctly repeat 22.60 sentences (out of 33).

We also compared the number of omissions at both times. The results show that children produced more omission errors at T1 (ẋ=39.75) compared to T2 (ẋ=22.05); t(19) = -2.04, *p =* .05.

**Figure 1:**
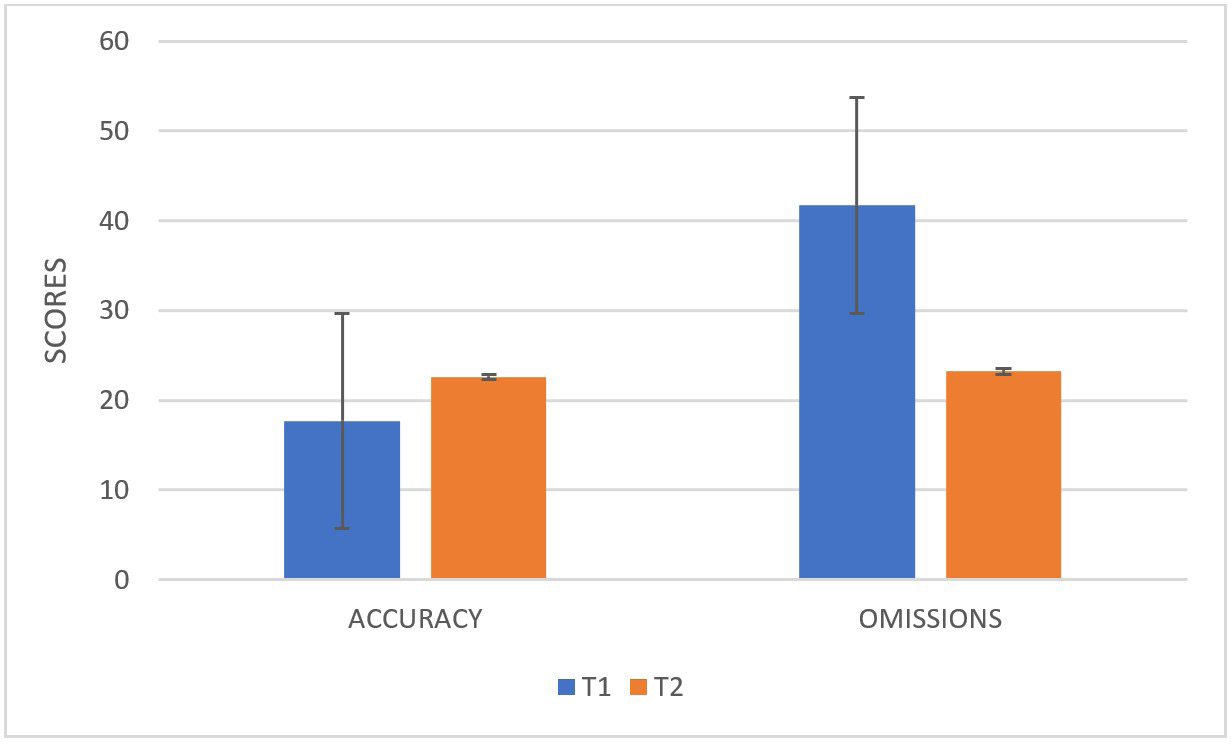
Differences in performance in the SRT at T1 and the SRT at T2

Figure 1 represents the changes in performance from T1 to T2.

### Does performance in the SRT at T1 predicts performance at T2?

**Figure 2:**
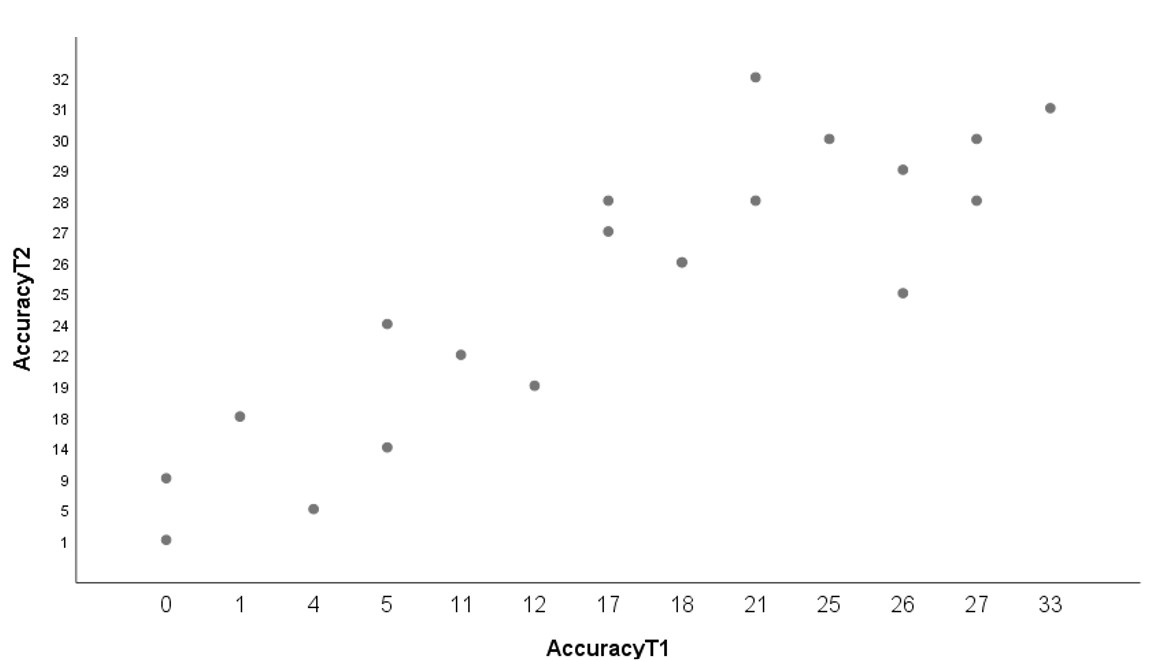
Linear regression between performance in the SRT at T1 and performance in the SRT at T2

The results of the regression analyses show a significant relationship between accuracy (number of items correctly repeated) in the SRT at T1 and accuracy in the SRT at T2 (F (1,18) = 43.160; *p* = .001, R^2^=.69) (see Figure 2).

This means that the 69% of the variance in performance at T2 can be explained just by performance at T1.

**Figure 3:**
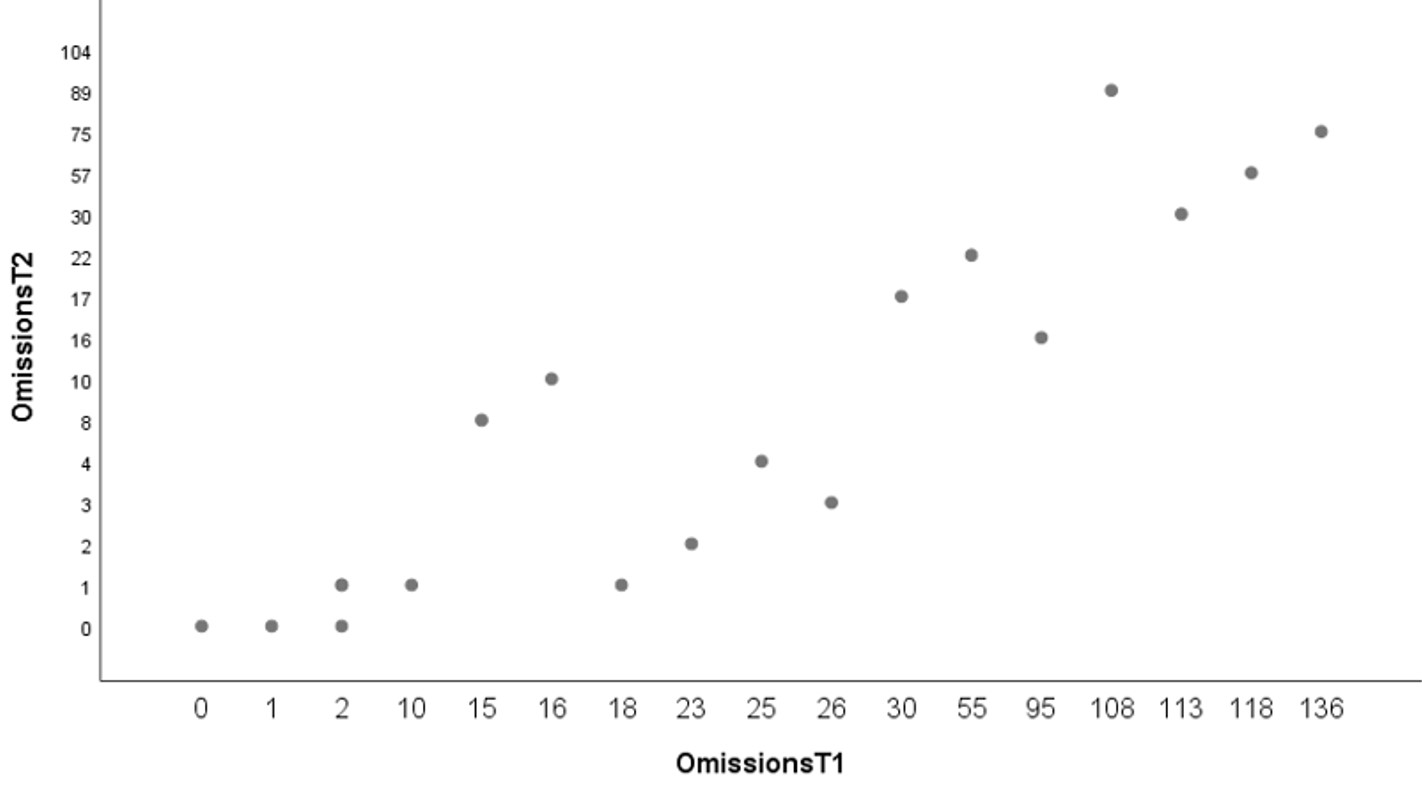
Linear regression between omissions in the SRT at T1 and omissions in the SRT at T2

In addition, we examined whether the omissions at T1 would predict omissions in the SRT at T2. Again, results show a significant relationship (F(1,18)= 8,44; *p* =.009, R^2^=.32). This means that omissions in the SRT at T1 predicts the number of omissions in sentence repetition task six months later (see Figure 3).

### Is performance in the SRT at T1 related to performance in other language measures, such as the NWR and the Merrill-Palmer-R at the same time?

Results of the Pearson correlations show that accuracy in the SRT at T1 is related to accuracy in the NWR (r = .802; *p* = .001). In addition, accuracy in the SRT at T1 is related to the scores achieved in the Merrill-Palmer-R (r = .932; *p* = .001). That is, accuracy in the SRT at T1 is significantly related to accuracy when repeating nonwords and to a general linguistic level measured with a standardized test. Participants reaching low scores in the SRT at T1 attain low scores both in the NWR and in the Merrill-Palmer-R. Likewise, participants reaching high scores in the SRT at T1 attain high scores both in the NWR and in the Merrill-Palmer-R.

### Does performance in the SRT at T1 predict performance in a standardized language measure such as the Merrill-Palmer-R at T2?

**Figure 4:**
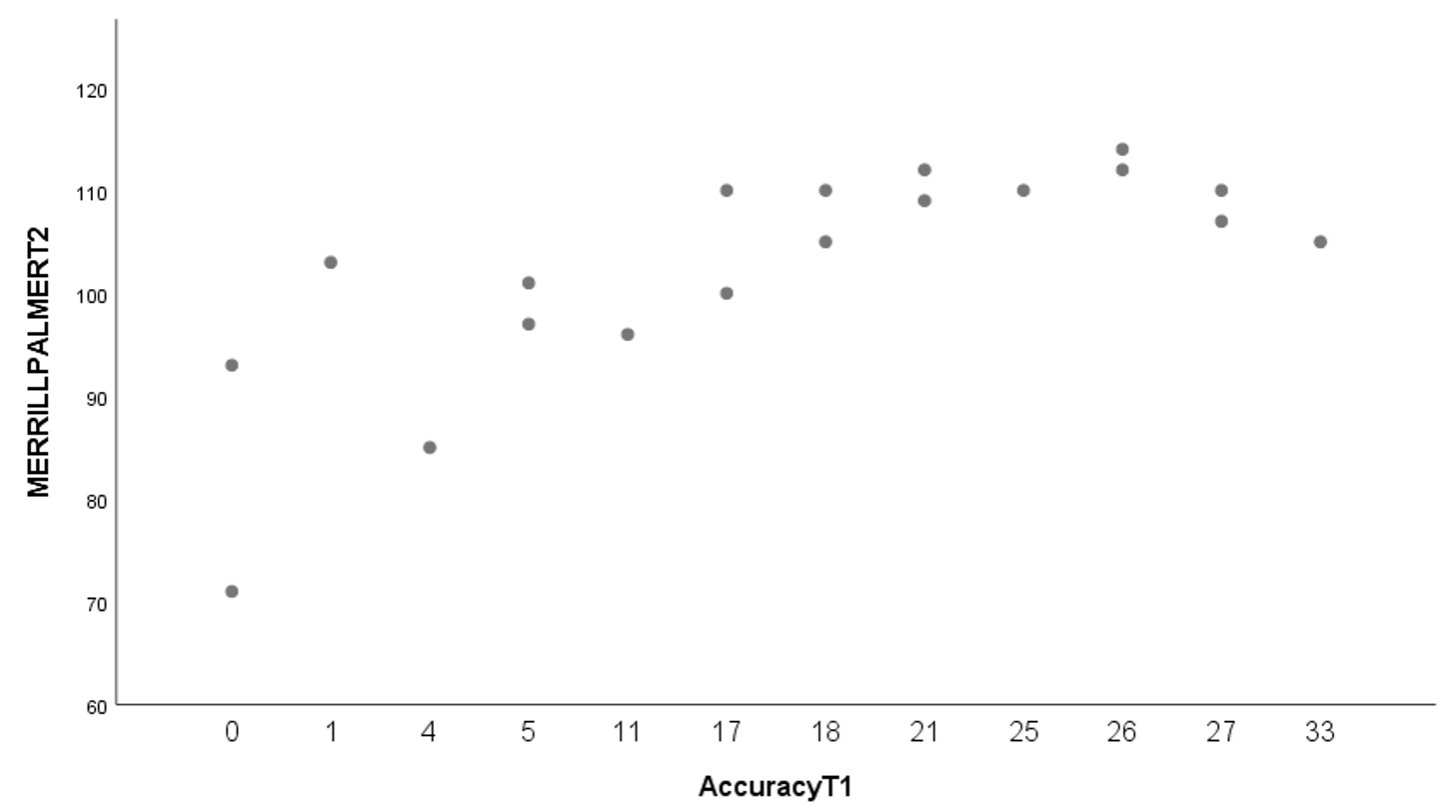
Linear regression between scores in the SRT at T1 and scores in the Merrill-Palmer-R at T2

Regression analyses showed a positive significant relationship between accuracy in the SRT at T1 and the score obtained in the Merrill-Palmer-R at T2 (F(1,17)=66,93; *p* =.001, R^2^= .80) (see Figure 4). That is, participants obtaining high scores in the SRT at T1 are likely to achieve high scores in the standardized test six months later.

## DISCUSSION

Previous evidence has shown that the SRTs are valid tasks for the early detection of language difficulties in children^(5)^. This cumulated research has motivated the development of this task in several languages with the aim of testing children, especially those aged above five years of age^(24)^. Developing SRTs for younger children is a complicated aim, given the wider variability in language abilities at early developmental stages. However, early detection is essential in order to offer children the necessary supports and opportunities to improve their communicative abilities, hence the need to design reliable and robust tests to identify children with language delays in these early stages.

In this longitudinal study we tested 20 monolingual Spanish-speaking children twice, with six months between assessments, in order to explore their ability at repeating sentences. This task has been developed to specifically assess children between 2 and 4 four years of age through 33 different sentences. Previous studies^(1)^ have shown that this task is adequate for this age group discriminating between different linguistic levels. The aim of this study was to explore the predictive validity of the task by examining the relationship between the scores in the SRT, the NWR and the Merrill-Palmer-R at T1 and T2.

The results obtained positively responded our research questions. The first question referred to the relationship between the scores in the SRT at T1 and T2. On the one hand, we have confirmed that the results in T1 strongly correlate with the scores at T2, therefore showing consistency among both applications. The higher scores obtained in the T2 also show that the task is sensitive to differences in children’s language abilities. From our point of view, this increase cannot be explained by any learning process of the materials employed, because six months between evaluations and the short age of the children seem to be sufficient not to consider this as an uncontrolled variable. However, it cannot be absolutely ruled out that the mere repetition of the task has played some subtle role. On the other hand, in line with^(1)^, the results also show that the number of omissions is significantly lower at T2 compared to T1. This suggests that this type of error is critical for scoring the task. In our view, the fact that there are less omissions in T2 compared to T1 reflects an increase in the children’s morphosyntactic abilities, and a decrease in the working memory demand (see^(11)^ for a discussion about the role of the working memory in the SRT)

The results obtained also positively responded to the second question, regarding predictability of the scores in T2 based on the results obtained in T1. Not only, accuracy in the SRT increased from T1 to T2 but also performance at T1 significantly predicts performance at T2.

The results discussed so far imply that the SRT is appropriate to evaluate young children language abilities; the SRT is sensitive to the language development; and the SRT can reasonably predict future performance of participants in the task.

The aims of the study also included the assessment of the relationship between the performance in the SRT and other linguistic tasks, such as the NWR and the Merrill-Palmer-R. Previous evidence has shown that SRT and NWR, despite slightly testing different language abilities, strongly correlate^(6,7,14,27)^. Therefore, to find a significant and positive correlation between both measures would have implied more consistency for the task assessed. The results in this respect are also clear-cut as we have observed a significant correlation between these scores. Indeed, the correlations were high as the relationship between the SRT and the NWR reached .80, and the correlation between the SRT and the Merrill-Palmer-R reached .93. In our view these results are relevant at supporting the validity of the task in this language.

Last, we wanted to examine the relationship between performance in the SRT at T1 and the standardized test at T2, finding a positive strong relationship between both tasks across time.

These results support the predictive validity of the SRT, as performance in this task predicts future performance in the same task as well as future performance in an already standardized linguistic test. This implies consistency as well as validity, so we consider that the Spanish SRT^(1)^ has been proved to be solid and robust tool for early language assessment.

However, we acknowledge some limitations of this study. On the one hand, the number of participants is not large and therefore the interpretation of the results has to be cautiously considered. Certainly, the results would have been strengthened with more children. On the other hand, the six months lasted from the first to the second administration is enough time for observing changes in the scores, but a third data taking would have offered interesting new evidence to better and further discuss the task administered.

## CONCLUSIONS

Results in this study can confirm that the SRT designed for children between 2 and 4 years^(1)^ shows adequate predictive validity values for its use at these early ages. This task is sensitive to the developmental changes shown by young children and allows to predict their language abilities measured at the same time with other tests, and even 6 months later.

Further studies will be necessary to continue exploring the validity of this early language assessment task. We believe that it may be interesting to reduce the number of items in the list of sentences that children have to repeat, shortening the test but maintaining the same reliability properties. In addition, studies including clinical populations would be necessary to test the sensitivity of this SRT in the detection of children following atypical trajectories in language development.

## Appendix A List of sentences and their translation into English

1. Mamá come (Mom eats)

2. Papá dice hola (Daddy says hello)

3. El coche es azul (The car is blue)

4. Es bonito el gato (Is beautiful the cat) (in Spanish the pronoun drop is correct)

5. Tienes que comer ya (You) have to eat just now)

6. Mi silla no es roja (My chair is not red)

7. ¿Qué mira tu papá? (What is your daddy looking at?)

8. La niña va al parque (The girl goes to the park)

9. El oso come mucha miel (The bear eats much honey)

10. Pon el pan en la mesa (Put the bread on the table)

11. Ahora vamos a coger los peces. (We are going to take the fishes now)

12. Los niños son muy guapos (The children are very Smart)

13. Aquí se sentó Ana (Ann sat here)

14. No quiero que me saques (I don’t want you take me out)

15. Esa señora come pan y jamón (That lady eats bread and ham)

16. Luis quiere leche y galletas para merendar (Luis wants milk and cookies for snack)

17. Hoy estará el abuelo (The grandaddy will be today)

18. Dice mamá que cojas el babi (Mommy says to you to take the dress)

19. Aquí hay más zumo que en tu casa (Here there is more juice than in your house)

20. Vamos al cole en el coche de papá (We go to school in daddy’s car)

21. Cuando quieras nos vamos a casa. (When you want we go home)

22. La niña llora porque quiere agua (The girl cries because she wants wáter)

23. Todos querían una galleta(Everybody want a cookie)

24. No deberías ir al patio (You shouldn’t go to the park)

25. El niño tiene que comer una pera (The boy has to eat a pear)

26. Esa niña lleva los zapatos que me gustan (That girl is wearing the shoes I like)

27. Ven a verme cuando salgas del cole (Come to see me when you leave school)

28. Se cayó al suelo y se hizo mucho daño (He fell down and feel a lot of pain)

29. Los niños son más altos que las niñas (The boys are taller than the girls)

30. El chocolate no te gusta pero a mí sí. (You don’t like chocolate but I do)

31. Si hace calor irás a la playa (If it is warm you will go to the beach)

32. ¿Cuándo se ha caído el niño? (When have the boy fallen down?)

33. Las niñas que llevan el vestido son mis amigas (The girls who are wearing the dress are my friends)
