# ERRATUM: A Sentence Repetition Task in Spanish language: a valid tool for early language assessment

**DOI:** 10.1590/2317-1782/e20230028en

**Published:** 2026-02-16

Due to an honest mistake by the authors, the article “A Sentence Repetition Task in Spanish language: a valid tool for early language assessment” (DOI https://doi.org/10.1590/2317-1782/20232022164en), present in CoDAS 2023;35(5):e20220164, was published with the incorrect financial support reference number.

**On page 1, in the "Financial support" section, where it reads:**

PDC2022-133484-I00

**It should read:**

PID2021-123907NB-I00

The authors apologize for the error.

